# Parental-couple well-being and relationship stability four years following childbirth: a mixed-methods study

**DOI:** 10.1186/s12978-026-02469-9

**Published:** 2026-10-09

**Authors:** Åsa Gamgam Leanderz, Margaretha Larsson, Maria Henricson, Stina Thorstensson, Caroline Bäckström

**Affiliations:** 1 School of Health Sciences, University of Skövde, Post Box 408, Skövde, S-541 28 Sweden; 2 Research School, School of Health and Welfare, Jönköping University, Jönköping, S-541 28 Sweden; 3 FamCeH, School of Health Sciences, University of Skövde, Skövde, S-541 28 Sweden; 4 Jönköping Academy, School of Health and Welfare, Jönköping University, Jönköping, S-551 11 Sweden; 5 Department of Caring Science, University of Borås, Borås, SE-501 90 Sweden

**Keywords:** Existential, Midwife, Parental Couple, Pregnancy, Parenthood, Sense of Coherence, Transition

## Abstract

**Objective:**

This study aimed to explore parents’ well-being and relationship stability during pregnancy and four years following childbirth.

**Background:**

Becoming a parent is a life event that challenges the parental couple and influences their relationship and well-being.

**Methods:**

This mixed-methods exploratory cohort study included 381 parents, followed longitudinally from pregnancy Week 25 to four years postpartum. Data were analysed using the logistic regression of survey data, Kaplan‒Meier estimates and qualitative content analysis. Survey instruments measured sense of coherence (a construct reflecting comprehensibility, manageability and meaningfulness), perceived social support and dyadic relationship quality, referring to the primary relationship between two cohabiting, unrelated adults.

**Results:**

After four years, 94% of the parental couples remained in a stable relationship. Greater relationship stability was associated with higher levels of a sense of coherence, stronger perceived social support and higher dyadic relationship quality among both mothers and partners. By four years after childbirth (T6), almost six out of 10 parents (59%) reported that nothing negatively impacted their well-being, indicating a decrease in well-being among the remaining 41% of the parents. Parents experience multidimensional vulnerability in the parental-couple relationship, and their well-being was negatively affected by the unpredictability of everyday life.

**Conclusion:**

Parents’ ability to maintain a sense of coherence, access and receive social support and maintain a high-quality couple relationship is associated with their relationship stability four years after childbirth.

**Supplementary Information:**

The online version contains supplementary material available at https://doi.org/10.1186/s12978-026-02469-9.

## Background

The transition to parenthood is a challenging period for parental couples [1], involving inequalities in childcare and related tasks as well as difficulties in combining and balancing multiple roles and identities, such as the self, a parent and a partner [2]. A central challenge is the perceived loss of couplehood and individuality due to the emergence of a primary identity as a parent. Parental couples may also face strong social expectations to continue performing and contributing across multiple life domains beyond parenting [2]. Despite extensive research on the transition to parenthood, important gaps remain. Previous studies have largely examined psychological, social or relational aspects separately, while the interplay between existential well-being, a sense of coherence and dyadic relationship quality over time remains insufficiently understood. This limits the understanding of how these dimensions jointly influence long-term relationship stability and parental well-being. In the current study, *relationship stability* is used as an umbrella term for *marital stability*. According to Kluwer [3], *marital stability* generally refers to whether spouses stay together. In Sweden, it is common to live in the same household in a marriage-like state without being married (i.e. cohabitation). Among Swedish individuals aged 20 or older, 1.3 million cohabited in 2011. This figure corresponds to 18.3% of the people in that group, the highest figure of all European Union countries [4].

Existential aspects of the transition to parenthood are closely intertwined with changes in parental relationships and perceptions of the meaning of life. According to Prinds et al. [5], this transition may evoke existential vulnerability, that is reflections on meaning, purpose and human vulnerability can contribute to a more authentic life experience [6]. Such reflections can enhance the experience of relational coherence [7] and are associated with relationship stability, which is also shaped by individual personality factors [8].

Marital satisfaction declines from pregnancy onwards and does not fully recover during the first postpartum year [9]. Simultaneously, sensuality and sexuality remain central dimensions of the parental relationship [10], while communication about intimacy is reshaped during this period [11]. These relational changes highlight the need to understand the factors that support sustained relationship quality.

Existential well-being reflects individuals’ perceptions of who they are, what they do, why they do it and where they belong, extending beyond the self towards others and the surrounding world [12]. It is a resource-oriented construct [13], and attaining it is a fundamental human motivation [14]. During pregnancy, existential well-being is strongly associated with a high sense of coherence (SOC) [15]. However, how existential well-being and SOC interact with relationship dynamics across the transition to parenthood and the early years of childrearing remains unclear.

Some studies suggest that parental couples may experience augmented relational functioning during this transition [16, 17]. For example, changes in SOC and perceived social support from pregnancy to six months postpartum have been linked to a higher perceived quality of the dyadic relationship (QDR) six months after childbirth [17]. Conversely, lower or declining SOC and perceived QDR after childbirth have been associated with parental-couple separation within two years postpartum [7].

Antonovsky [18] conceptualised a sense of coherence as a relatively stable disposition, although it may also be understood as dynamic in its interaction with internal and external stressors. Factors that may explain why some parental couples experience a more positive transition include adaptive processes as well as personal and situational characteristics [3]. *Adaptive processes* refer to how couples manage and cope with difficulties, while personal and situational characteristics can influence both the impact of the transition and how couples adapt to it [19]. First-time parents often rely on their social networks to obtain social support, which enhances their understanding of how to prepare for parenthood and makes the upcoming role feel more concrete and real [20, 21]. *Social support* refers to the relational ties between individuals [22] and is identified as an important factor shaping the experience of the transition to parenthood [23], often serving as a buffer during stressful periods [22]. Furthermore, low levels of perceived social support during pregnancy and the first six months postpartum have been associated with parental separation [7]. The mutual support provided within the couple can itself be understood as a form of social support [24]. In this context, it is also important to consider broader household conditions when studying the transition to parenthood [11], as changes in employment status may influence relationship dynamics [25]. In addition, the equal sharing of parental leave has been linked to better-perceived couple relationship quality among fathers, but not mothers, at 18 months postpartum [26]. Professional support during pregnancy has been shown to strengthen parental couples’ relationships [27]. In Sweden, pregnant women usually attend public antenatal care and receive regular check-ups with a midwife starting from weeks 5‒7 of pregnancy, with follow-up visits recommended for 6‒16 weeks after delivery. The coverage rate for registered childbirths is 98.7% [28]. After childbirth, the parents regularly visit the Child Healthcare (CHC) centre with their newborn baby until the child is six years old.

The transition to parenthood requires major adjustments to parents’ daily lives and presents challenges that demand considerable personal resources. Parents often report feeling insufficiently informed about this demanding life stage [24, 29], highlighting the importance of professionals such as midwives and CHC nurses knowing how to support parents’ well-being and couple relationships during this transition. There is still limited knowledge about the changes in the existential aspects of well-being that occur when becoming a parent [30], and the stability of couples’ relationships during the transition to parenthood needs further investigation. Therefore, this study explored parents’ well-being and relationship stability during pregnancy and up to four years following childbirth.

## Methods

This exploratory quantitative study employs mixed methods and is based on primary analyses. It is part of the longitudinal cohort The Study of Parental Support, which evaluates first-time mothers’ and partners’ perceptions of professional parental support during childbearing [7, 17, 21, 27, 31].

Participants were recruited on two occasions, first within antenatal care (gestational Week 25) (T1) and then within postnatal care (first week after childbirth) (T2) between September 2014 and April 2016 in a southwestern county of Sweden with 280,000 inhabitants. Initially, respondents were recruited only via antenatal care (T1). However, there were difficulties with recruiting that way, which meant that a supplementary ethics application expanded the possibility of recruiting via postnatal care (T2). Accordingly, some informants did not answer T1 because they were recruited into the study at T2. The study’s geographic areas are both rural and suburban and are, hence, representative of the ordinary Swedish population. The labour ward at the county’s only hospital has about 2,700 childbirths per year. Ante- and postnatal care in Sweden consists of midwifery units and a CHC centre.

**Fig. 1:**
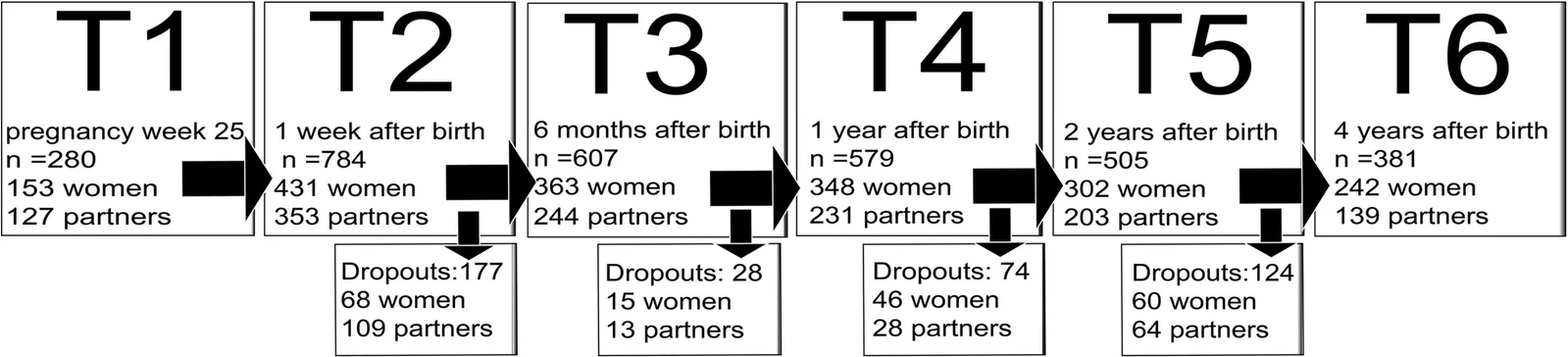
Flow chart with the response rate represented by n. Recruitment took place on two occasions (T1 and T2). The participants did not specify any reason for their dropping out

The inclusion criteria for this study were first-time mothers (termed *mothers*) and partners who were expecting or had given birth to a singleton infant. The exclusion criteria were non-Swedish-speaking mothers and partners and the birth of multiples (e.g. twins or triplets). This study used partners as the collective name for fathers and co-mothers as the non-birthing parent, regardless of marital or cohabitation status. This study included two co-mothers. For this research, data were collected with questionnaires at six stages of time (T): Week 25 of pregnancy (T1), one week (T2), six months (T3), one year (T4), two years (T5) and four years (T6) after childbirth. The number of participants and the response rate for each questionnaire are presented in Fig. 1. The parents accessed information about the study in both written and verbal formats from the midwives. After agreeing to participate, the subjects were emailed a hyperlink to the web-based questionnaires in EvaSys (Evaluation System) Version 7.0. Three at each time point (T1–T6) were sent to the participants who had not completed the questionnaire. The participants did not receive any compensation.

In total, 381 participants answered T6 and were included in data analysis; 107 informants (71 mothers and 36 partners) completed all questionnaires for the entire study period.

### Variables

The current study included socio-demographic questions such as age, education, country of birth, marital status, occupation, additional births and length of the parental-couple’s relationship. A question on parents’ participation in professional preparatory support was included as well as the perceived state of their household finances (1 = Strained; 2 = Adequate; 3 = Good; 4 = Very good). Table 1 also includes three validated instruments ‒ Sense of Coherence (SOC-13) [32], Multidimensional Scale of Perceived Social Support (MSPSS) [20], and Quality of Dyadic Relationship (QDR) [33] ‒ as well as questions concerning well-being.

**Table 1:** Baseline characteristics – SOC-13, MSPSS and QDR36 – at various time points (T1–T6)

	Mothers	Partners
	Mean (SD)	Mean (SD)
Participant, n	242	139
Age at birth of the first child (yrs.)	27.7 (4.2)	30.4 (6.3)
Education T6 (yrs.)	14.4 (3.0)	14.0 (2.6)
Country of birth of the participant, n (%)		
Sweden	228 (95.8)	132 (95.7)
Norway, Denmark, Finland, Iceland	1 (0.4)	1 (0.7)
Another country in Europe	3 (1.3)	3 (2.2)
Another country outside Europe	6 (2.5)	2 (1.4)
Still in the same parental-couple relationship T6, as 4 years ago		
(yes)	228	133
(no)	13	6
Marital status T6, n		
Married	123	68
Cohabitating	108	66
Separate accommodations	2	0
Single	9	4
Occupation T6, n (%)		
Parental leave	61 (12.5)	17 (4.0)
Employee	163 (33.3)	123 (28.7)
Studying	23 (4.7)	3 (0.7)
Sick leave	6 (1.2)	2 (0.5)
Unemployed	5 (0.1)	0 (0)
Other	3 (0.6)	2 (0.5)
Perceived economy, range 1‒4		
T1	2.8 (0.7)	2.9 (0.7)
T2	2.8 (0.7)	3.0 (0.7)
T3	2.4 (0.7)	2.8 (0.7)
T4	2.4 (0.8)	2.5 (0.8)
T5	2.7 (0.8)	2.8 (0.8)
T6	2.8 (0.9)	2.8 (0.8)
Additional births, (yes) n		
T4	2	0
T5	66	43
T6	175	97
Length of parental-couple relationship T6 (yrs)	9.2 (3.1)	9.7 (3.7)
Separation from partner T4‒T6 (yes), n (%)		
T4	7 (1.4)	3 (0.7)
T5	13 (2.7)	9 (2.1)
T6	13 (5.5)	6 (4.3)
If something occurred that affected everyday well-being negatively T2‒T6 (no), range 1‒3		
T2	2.91 (0.36)	2.94 (0.28)
T3	2.79 (0.48)	2.89 (0.39)
T4	2.82 (0.48)	2.84 (0.43)
T5	2.71 (0.55)	2.74 (0.58)
T6	2.64 (0.62)	2.58 (0.65)
Perceived professional preparatory support T3, range 1–4 (very satisfying)	3.22 (0.65)	3.20 (0.56)
Sense of Coherence (SOC-13) index T1‒T6, range 13–91		
T1	66.3 (12.2)	69.7 (10.5)
T2	71.3 (10.8)	72.0 (10.4)
T3	70.7 (11.9)	71.1 (11.4)
T4	70.3 (12.7)	71.1 (11.6)
T5	70.1 (12.4)	70.5 (11.7)
T6	69.1 (12.3)	70.9 (11.7)
Mean		
Mean SOC-13 index T2–T3	71.0 (10.4)	71.8 (10.0)
Mean SOC-13 index T2–T4	71.1 (10.6)	71.5 (10.0)
Mean SOC-13 index T2–T5	71.2 (10.6)	70.9 (10.3)
Mean SOC-13 index T2–T6	70.2 (10.6)	71.3 (11.0)
Change		
Change in SOC-13 index between T2–T3	0.3 (8.9)	1.9 (8.3)
Change in SOC-13 index between T2–T4	1.0 (9.2)	1.3 (8.9)
Change in SOC-13 index between T2–T5	0.9 (9.3)	1.9 (8.8)
Change in SOC-13 index between T2–T6	1.7 (9.6)	1.9 (8.5)
Multidimensional Scale of Perceived Social Support (MSPSS) index T1–T6, range 12–84		
T1	73.9 (10.3)	71.4 (11.0)
T2	79.5 (6.7)	77.1 (8.9)
T3	74.7 (10.5)	71.5 (10.8)
T4	73.6 (11.5)	71.1 (12.1)
T5	72.7 (12.0)	69.6 (12.5)
T6	71.3 (13.1)	70.0 (13.0)
Mean		
Mean MSPSS index T2–T3	77.2 (7.7)	74.4 (8.7)
Mean MSPSS index T2–T4	76.1 (8.5)	73.4 (9.3)
Mean MSPSS index T2–T5	75.2 (9.2)	72.7 (9.4)
Mean MSPSS index T2–T6	73.8 (9.4)	72.9 (10.0)
Change		
Change in MSPSS index between T2–T3	4.5 (7.7)	5.9 (8.4)
Change in MSPSS index between T2–T4	5.7 (8.7)	6.5 (9.5)
Change in MSPSS index between T2–T5	6.5 (9.2)	7.8 (10.0)
Change in MSPSS index between T2–T6	7.3 (10.3)	7.1 (9.8)
Quality of Dyadic Relationship (QDR36) index T1–T6, range 5–30		
T1	24.6 (2.3)	24.5 (2.1)
T2	25.4 (2.1)	25.2 (2.1)
T3	25.4 (2.0)	25.2 (2.1)
T4	23.9 (2.6)	23.9 (2.7)
T5	23.7 (2.8)	23.8 (2.7)
T6	22.6 (3.5)	23.1 (2.8)
Mean		
Mean QDR36 index T2–T3	24.7 (2.1)	24.5 (2.3)
Mean QDR36 index T2–T4	24.4 (2.2)	24.3 (2.3)
Mean QDR36 index T2–T5	24.3 (2.2)	24.1 (2.4)
Mean QDR36 index T2–T6	23.9 (2.4)	24.1 (2.3)
Change		
Change in QDR36 index between T2–T3	1.4 (2.0)	1.4 (1.9)
Change in QDR36 index between T2–T4	4.3 (2.6)	1.6 (1.7)
Change in QDR36 index between T2–T5	2.1 (2.3)	2.1 (1.9)
Change in QDR36 index between T2–T6	2.5 (2.6)	2.4 (2.8)

T1 = Pregnancy Week 25; T2 = 1 Week After Birth; T3 = 6 Months After Birth; T4 = 1 Year After Birth; T5 = 2 Years After Birth and T6 = 4 Years After Birth

The SOC-13 scale consists of 13 items. Responses were made on a Likert scale ranging from 1 (very often) to 7 (very seldom). The SOC-13 construct entails a general position of the world, conceiving of it as manageable (five items), meaningful (four items) and comprehensible (four items) [34]. *Manageability* refers to a person’s capacity to comprehend what is happening around them, assess how much control they have over a situation, either independently or with support from people in their social network, and identify a sense of meaning in the experience [35]. On the SOC-13, each item summed up enables an index with a score ranging from 13 to 91 [36]. A higher number on the SOC-13 scale indicates a strong SOC-13 [35]. Relatively speaking, the SOC-13 instrument appears to be psychometrically sound, valid, reliable and applicable cross-culturally, measuring how individuals manage stressful situations and stay well [37]. The SOC-13 instrument also has been validated for pregnant women [36]. The SOC-13 seems comparatively stable over time, leastwise for individuals with a high SOC but not as firm, as Antonovsky postulated. However, the SOC-13 tends to increase with ag e. The SOC-13 scale showed high internal consistency in previous research [37], with Cronbach’s alpha [38] ranging from 0.70 [39] to 0.92 [40] in 127 studies using the SOC-13 [37]. Furthermore, the test‒retest reliability of the SOC-13 ranges from 0.69 [41] to 0.72 [42], as stated by Eriksson and Lindström [37].

The MSPSS scale comprises 12 items. Responses were made on a Likert scale ranging from 1 (very strongly disagree) to 7 (very strongly agree). The three dimensions of the MSPSS are family, friends and significant others, with four items each. Each dimension contains scores between 4 and 28. The MSPSS index scores range between 12 and 84, and a higher score indicates higher perceived social support. The MSPSS index and the dimensions were calculated and used for regression analyses (Tables 1, 2 and 3). The MSPSS was developed as a self-reported measure of subjectively assessed social support [22] and is psychometrically sound [22] and validated in pregnant women [34]. The MSPSS [22] has been translated, adapted and evaluated for use in Swedish contexts. The psychometric evaluation was conducted on a main sample of women with hirsutism and nursing students. The results showed high internal consistency and confirmed validity and reproducibility [43].

**Table 2:** Simple logistic regression analysis of relationship stability T6, at different time points (T1–T6)

	Mothers	Partners
	OR (95% CI)	OR (95% CI)
Participant, n	242	139
Age at birth of the first child	1.10 (0.93–1.29)	1.00 (0.86–1.14)
Education T6 (yrs)	**1.18** (1.03–1.36)*	**1.46** (1.09–1.97)*
Country of birth of the Participant T6 (Sweden)	1.40 (0.66–3.00)	a
Marital status T6 range 1–4 (married)	a	a
Occupation T6		
Parental-leave T6	4.29 (0.55–33.67)	a
Employee T6	0.64 (0.17–2.38)	1.46 (0.16–13.33)
Studying T6	0.27 (0.16–10.33)	a
Sick-leave T6	0.27 (0.03–2.49)	**0.04** (0.002–0.70) *
Unemployed T6	a	-
Other T6	a	a
Perceived economy		
T1	2.78 (0.89–8.67)	1.72 (0.29–10.20)
T2	**3.53** (1.49–8.40)**	**4.34** (1.38–13.65)*
T3	1.87 (0.84–4.18)	0.93 (0.30–2.88)
T4	**2.49** (1.09–5.70)*	1.02 (0.32–3.26)
T5	2.07 (0.89–4.81)	**3.15** (1.12–8.82)*
T6	1.89 (0.996–3.59)	1.64 (0.58–4.67)
Planned pregnancy 2 years ago T5 (yes)	0.77 (0.10–6.08)	0.97 (0.12–8.00)
Additional births, range 1–3 (1, 2 and > 2 births)		
T4	a	a
T5	1.88 (0.34–10.46)	a
T6	**4.33** (1.13–16.51)*	a
Length of couple relationship T6	1.32 (0.96–1.83)	a
If something occurred that affected every-day well-being negatively T2–T6 (no)		
T2	0.90 (0.16–5.02)	a
T3	1.87 (0.67–5.18)	a
T4	1.80 (0.78–4.19)	a
T5	1.72 (0.62–4.77)	**2.89** (1.07–7.81)*
T6	1.73 (0.83–3.62)	**3.19** (1.11–9.12)*
Professional Preparatory Support		
Attending Professional Preparatory Support with Partner T5 (yes)	0.50 (0.10–2.49)	a
Perceived Professional Preparatory Support T3	1.96 (0.84–4.57)	3.11 (0.80–12.02)
SOC-13		
SOC-13 index T1	1.03 (0.96–1.10)	1.01 (0.91–1.11)
SOC-13 index T2	1.01 (0.96–1.07)	1.00 (0.92–1.10)
SOC-13 index T3	**1.05** (1.00–1.09)*	1.00 (0.94–1.07)
SOC-13 index T4	1.03 (0.99–1.07)	0.99 (0.92–1.07)
SOC-13 index T5	1.00 (0.95–1.06)	1.07 (0.99–1.15)
SOC-13 index T6	**1.06** (1.02–1.11)**	1.03 (0.96–1.09)
Mean		
Mean SOC-13 index T2–T3	1.03 (0.98–1.09)	0.998 (0.91–1.09)
Mean SOC-13 index T2–T4	1.03 (0.98–1.09)	0.99 (0.90–1.09)
Mean SOC-13 index T2–T5	1.02 (0.96–1.09)	1.02 (0.91–1.14)
Mean SOC-13 index T2–T6	1.02 (0.96–1.09)	1.01 (0.90–1.14)
Change		
Change in SOC-13 index between T2–T3	0.97 (0.91–1.03)	1.03 (0.88–1.19)
Change in SOC-13 index between T2–T4	0.97 (0.91–1.03)	1.10 (0.96–1.26)
Change in SOC-13 index between T2–T5	1.01 (0.92–1.09)	**0.76** (0.60–0.96)*
Change in SOC-13 index between T2–T6	1.06 (0.995–1.12)	1.03 (0.93–1.14)
MSPSS		
MSPSS index T1	0.92 (0.81–1.05)	1.06 (0.98–1.14)
MSPSS index T2	1.02 (0.94–1.11)	**1.11** (1.02–1.21)*
MSPSS index T3	0.98 (0.91–1.05)	**1.09** (1.01–1.17)*
MSPSS index T4	0.97 (0.91–1.03)	1.06 (0.998–1.12)
MSPSS index T5	0.92 (0.83–1.02)	1.06 (0.997–1.13)
MSPSS index T6	1.01 (0.97–1.05)	**1.06** (1.00–1.12)*
Mean		
Mean MSPSS index T2–T3	0.96 (0.86–1.06)	**1.10** (1.01–1.20)*
Mean MSPSS index T2–T4	0.95 (0.86–1.05)	**1.09** (1.01–1.17)*
Mean MSPSS index T2–T5	0.95 (0.85–1.06)	1.07 (0.99–1.16)
Mean MSPSS index T2–T6	0.95 (0.86–1.06)	1.07 (0.99–1.16)
Change		
Change in MSPSS index between T2–T3	**1.17** (1.05–1.30)**	0.995 (0.88–1.12)
Change in MSPSS index between T2–T4	**1.10** (1.00–1.20)*	0.998 (0.90–1.11)
Change in MSPSS index between T2–T5	**1.12** (1.02–1.23)*	1.02 (0.91–1.15)
Change in MSPSS index between T2–T6	0.94 (0.87–1.01)	1.01 (0.92–1.10)
QDR36		
QDR36 index T1	1.34 (0.87–2.05)	1.09 (0.55–2.15)
QDR36 index T2	1.11 (0.78–1.59)	1.19 (0.75–1.89)
QDR36 index T3	**1.37** (1.08–1.74)**	1.17 (0.87–1.58)
QDR36 index T4	1.18 (0.96–1.44)	1.29 (0.93–1.78)
QDR36 index T5	**1.26** (1.01–1.56)*	**1.41** (1.00–1.99)*
QDR36 index T6	**1.37** (1.14–1.66)***	0.59 (0.29–1.19)
Mean		
Mean QDR36 index T2–T3	1.19 (0.80–1.77)	1.11 (0.72–1.72)
Mean QDR36 index T2–T4	1.20 (0.84–1.73)	1.21 (0.80–1.85)
Mean QDR36 index T2–T5	1.13 (0.71–1.81)	1.79 (0.86–3.73)
Mean QDR36 index T2–T6	1.66 (0.87–3.17)	1.29 (0.56–2.95)
Change		
Change QDR36 index T2–T3	**0.71** (0.51–1.00)*	1.01 (0.56–1.83)
Change QDR36 index T2–T4	**0.72** (0.53–0.96)*	0.66 (0.36–1.19)
Change QDR36 index T2–T5	0.82 (0.56–1.20)	0.56 (0.30–1.04)
Change QDR36 index T2–T6	1.03 (0.72–1.46)	0.11 (0.01–1.28)

OR–Values with a Significant *P*-value, Marked in Bold. *p-value ≤ 0.05; ** *p*-value ≤ 0.01; *** *p*-value ≤ 0.001 and a = Did Not Converge

**Table 3:** Multiple logistic regression analysis of relationship stability at different time points (T2–T6)

INDEX	Mothers	Partners	Cox and Snell	Naegel- kerke	
	OR (95% CI)	OR (95% CI)			
Model 1, T1			**—**	**—**	
No Variables for Multiple Regression	**—**	**—**			
Model 2, T2			0.047	0.154	
Perceived Economy	**2.49** (1.32–4.69)**	1.91 (0.78–4.66)			
MSPSS Family	**—**	**2.14** (1.08–4.22)*			
Model 3, T3			0.052	0.171	
SOC Manageability	1.26 (0.39–4.02)	**—**			
SOC Meaningfulness	**—**	**—**			
MSPSS Family	**—**	1.90 (0.72–5.02)			
MSPSS Significant Others	**—**	1.18 (0.41–3.43)			
QDR36 Satisfaction	**5.99** (1.57–22.91)**	**—**			
QDR36 Consensus	0.73 (0.31–1.77)	**—**			
Model 4, T4			0.049	0.158	
Perceived Economy	1.71 (0.75–3.93)	**—**			
MSPSS Significant Others	**—**	1.79 (0.83–3.83)			
QDR36 Consensus	**—**	**—**			
QDR36 Satisfaction	**2.83** (1.12–7.18)*	**6.31** (1.15–34.58)*			
Model 5, T5			0.053	0.221	
Perceived Economy	**—**	3.12 (0.92–10.62)			
QDR36 Index	a	1.11 (0.33–3.70)			
Model 6, T6			0.068	0.259	
Perceived Economy	**—**	**—**			
SOC Index	**2.75** (1.02–7.45)*	**—**			
MSPSS Index	**—**	a			
QDR36 Index	**2.32** (1.06–5.08)*	**—**			

OR–Values with a Significant *P*-value, Marked in Bold. *p-value ≤ 0.05; ** *p*-value ≤ 0.01; *** *p*-value ≤ 0.001 and a = did not converge

The QDR36 instrument contains 36 questions and assesses the perceived quality of the *parental dyadic relationship*, defined as the primary relationship between two unrelated adults who live together [44]. Responses were made on a Likert scale that ranged from 1 (always disagree/never) to 6 (always agree/more often). The QDR instrument has five dimensions: consensus (11 items), cohesion (four items), satisfaction (11 items), sensuality (five items) and sexuality (five items). The QDR index’s score ranges between 5 and 30; a higher score indicates a higher perceived quality of the dyadic relationship. The QDR instrument is a modified version [33] of the DAS (Dyadic Adjustment Scale) [44], which was used across the transition to parenthood to assess stability and marital changes [45]. A psychometric evaluation was first conducted on a sample of Swedish parents six months after childbirth [33] and, second, on women and men living in long-term relationships [46]. The QDR is considered a valuable instrument for both sexes, with internal consistency, explanation of variance [33] and good validity and reliability [46].

To assess parental well-being in this study, a question was created with three response options: Has something occurred in your life that has affected your everyday well-being negatively since you filled out the previous survey (T2–T6)? The response options are 1: Yes, to a large extent, 2: Yes, to some extent and 3: No. Furthermore, a question was created to capture the parents’ descriptions of what affected their well-being: If you answered yes to the previous question, can you describe what has happened (T2–T6)? (written answers, with a maximum of 80 words at each time point).

### Analytical strategy

In this study, the quantitative data were analysed using the Statistical Package for the Social Sciences (SPSS) Version 26, whereas the qualitative data were analysed with summative content analysis inspired by Hsieh and Shannon [47]. The questionnaires were web based, and participants’ responses could be directly imported into SPSS. The acronym of the participant’s name was replaced by a number in the file used for analysis. The subjects’ identities were kept confidential, meaning that only the first and last authors had access to their identities. Values entered incorrectly were excluded from the analysis. The quantitative analyses, index, dimensions, mean and change score calculations were computed for the specific measures provided. Of the 381 participants eligible for analyses, 198 represented the same couple relationship, meaning that both the mother and partner living together at T2 answered at all the time points (T2–T6). Descriptive statistics (Table 1) and simple (Table 2 and Supplementary file 1), as well as multiple logistic regression analysis (Table 3), were performed separately to evaluate the factors associated with relationship stability during the first four years after childbirth for mothers and partners. The outcome variable relationship stability four years after childbirth (T6) was created based on this question: Do you still have a relationship with the partner you had a child with four years ago? The outcome variable was dichotomised to fit logistic regression (0 = not living together; 1 = living together). Each variable was analysed separately in a simple logistic regression model (Table 2).

Furthermore, univariate variables from the simple logistic regression (See Supplementary file 1) with a significant p-value (< 0.05) included in the multiple logistic regression analysis were named Model 1 to Model 6 (Model 1 describes pregnancy Week 25 = T1). The chosen variables for multiple analysis were the instruments’ dimensions or indexes (sense of coherence, social support and quality of the dyadic relationship) to evaluate whether these factors were related to relationship stability. Correspondingly, the financial variable was included based on previous research showing that perceived financial wellness is associated with a higher QDR quality of dyadic relationships among parents [17]. The results from the multiple logistic regression analysis models were presented as standardised values using Z-transformation before analysis to obtain variables at the same level, facilitating the interpretation of the results (Table 3). A consideration of significance was set to a p-value < 0.05.

To evaluate the robustness of the primary logistic regression findings, a sensitivity analysis was performed using Generalized Estimating Equations (GEE). Repeated measurements of SOC, MSPSS, and QDR obtained across five assessment waves (T2-T6) were included in the models. Participant ID was specified as the subject variable and time as the within-subject variable. A GEE model with a binomial distribution, logit link function, and independent working correlation structure was used to account for repeated measurements within subjects. Odds ratios (ORs), 95% confidence intervals (CIs), Wald χ² statistics, and p-values derived from the GEE models are presented in Table 4.

**Table 4:** Generalised Estimating Equations (GEE) longitudinal analysis of predictors for relationship stability across timepoints T2–T6

Variable	Mothers			Partners		
	OR (95% CI)	Wald χ²	*p*-value	OR (95% CI)		
SOC	0.997 (0.99–1.01)	0.512	0.47	**a**		
MSPSS	**0.99** (0.99-1.00)	4.032	0.045*	**a**		
QDR	**1.02** (1.01–1.04)	8.365	0.004**	**a**		
Age at birth of the first child	1.18 (0.98–1.43)	2.194	0.14	**a**		
Education T6 (yrs)	**1.21** (1.04–1.42)	6.890	0.01**	**a**		
Additional births, (yes) T4	**0.28** (0.05–1.52)	4.516	0.03*	**a**		

OR–Values with a Significant *P*-value, Marked in Bold. *p-value ≤ 0.05; ** *p*-value ≤ 0.01; *** *p*-value ≤ 0.001 and a = did not converge

**Fig. 2:**
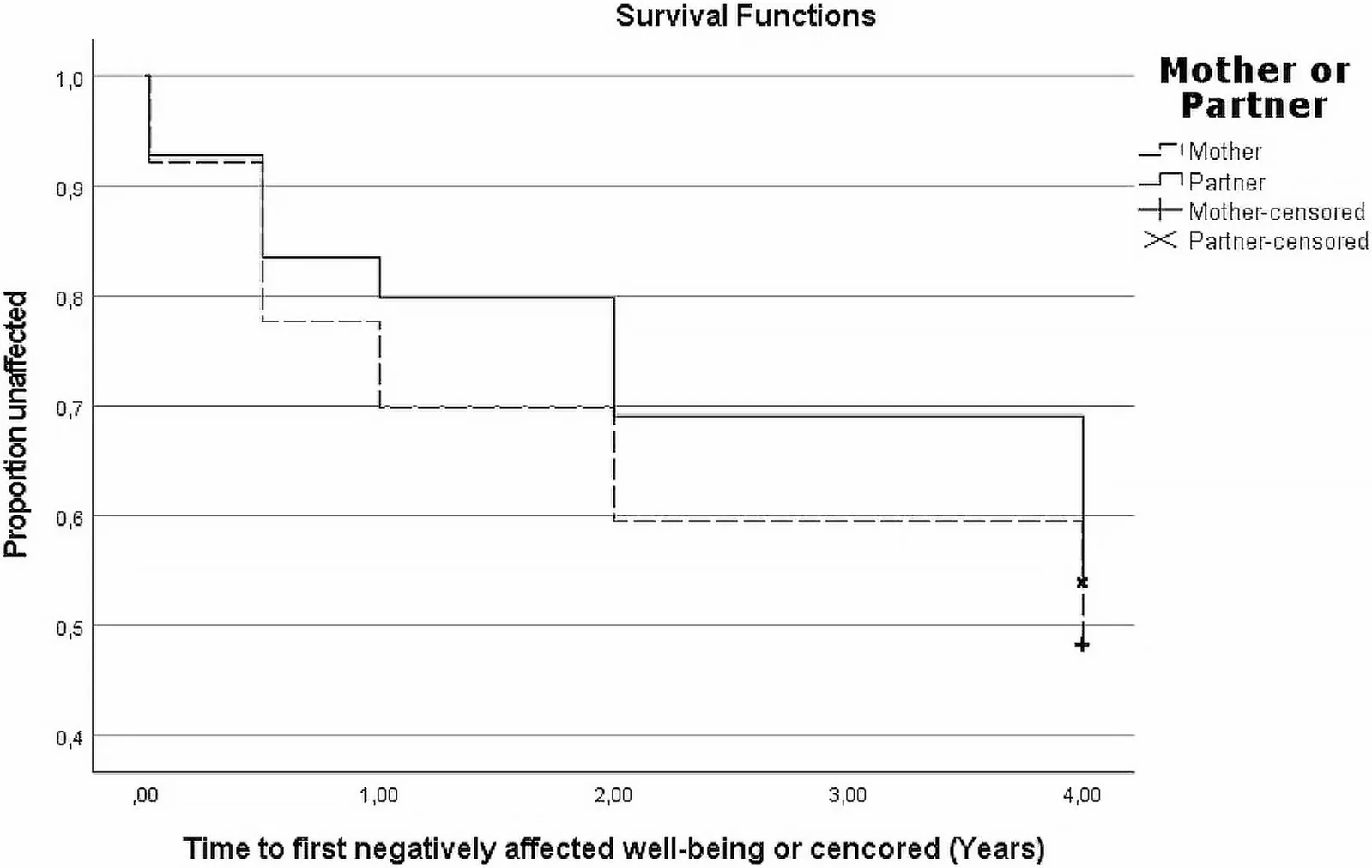
Kaplan–Meier survival analysis of first instance of negatively affected well-being for mother and partner (T2–T6)

**Fig. 3:**
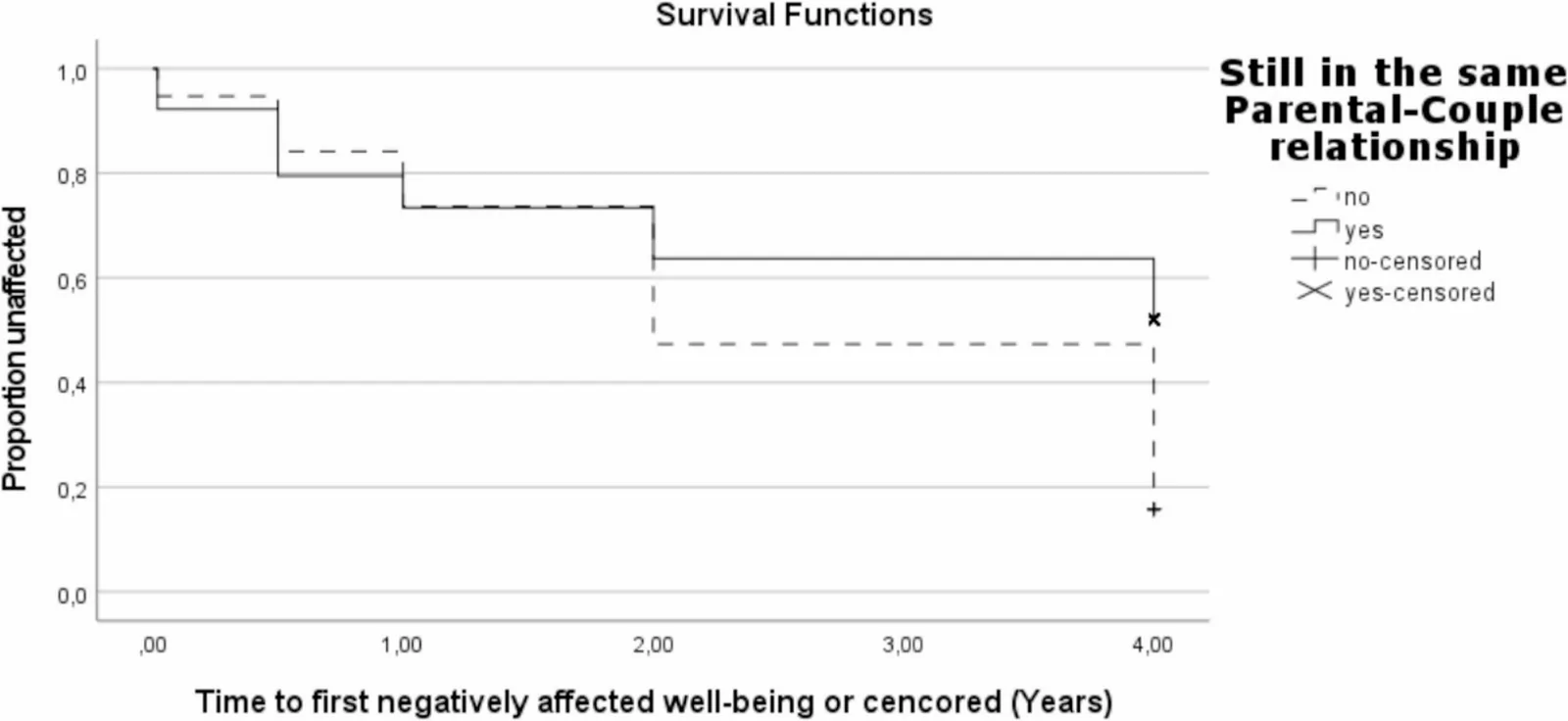
Kaplan–Meier survival analysis of first instance of negatively affected well-being for parents in a parental-couple relationship or separated (T2–T6)

To assess changes in well-being over time, Kaplan–Meier analyses were conducted to estimate the time to the first reported decline in well-being for mothers and their partners from one week (T2) to four years (T6) after childbirth (Fig. 2). The variable ‘Did something occur in your life that negatively affected your everyday well-being?’ was recoded into a binary format (yes/no). Separate Kaplan–Meier analyses were performed to identify changes in well-being for parents who had a stable relationship at four years and for those who experienced parental-couple separation between the first week (T2) and four years (T6) after childbirth (Fig. 3).

**Fig. 4:**
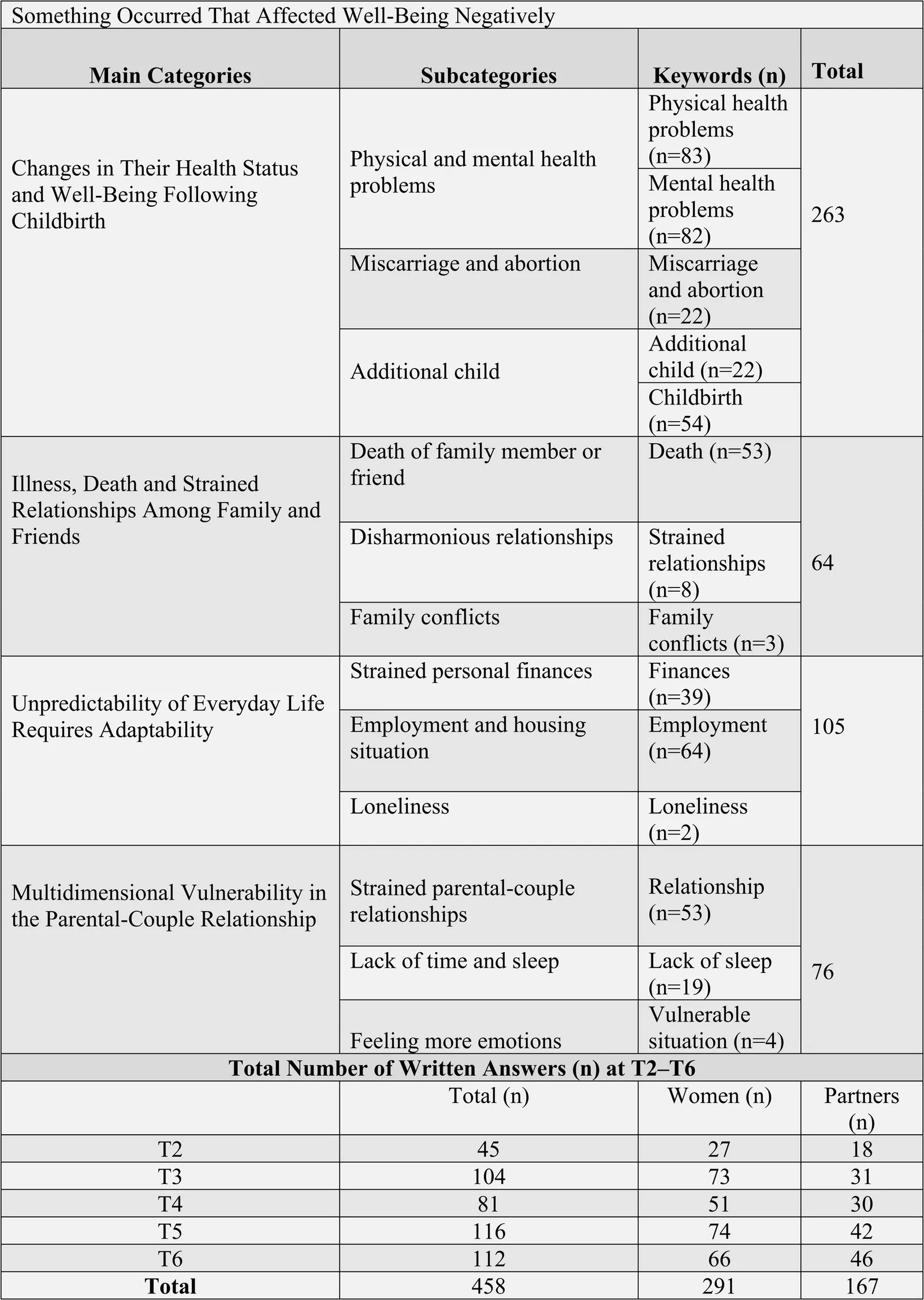
Results for the main categories, subcategories, keywords and qualitative analysis

The qualitative data analysis was inspired by summative content analysis [47], which entailed comparisons and the counting of keywords. The keywords were identified before and during data analysis and stem from a review of the literature or the interests of researchers [47]. The qualitative answers to the open-ended question were placed in a Word document sorted by time points (T2–T6), and the negatively affected well-being was expressed. This enabled an overview of the material, marking keywords and simplifying the counting of the keywords the informants described. All participants’ written texts were then printed and placed on a table to obtain an overview of the material. The participants’ written texts were read several times during this process. Counting was used to identify data patterns and contextualise codes. This allowed an interpretation of the context associated with using the word or phrase. The summative approach surpasses mere word counts to include latent content analysis [47]. Subsequently, the meanings of the sentences were compared, grouped into categories based on their content and given names by the first author (a PhD student and registered midwife). The written texts were then read repeatedly for several weeks, and their meanings were reflected upon and regrouped. In addition, all the answers (*n* = 458) were assembled on five written sheets corresponding to each time point (T2–T6). The answers on the sheet were sorted into two columns corresponding to answers on where well-being had been affected to some extent and whether that well-being was affected mainly. The answers were also sorted into mothers and partners. Afterward, the keywords were highlighted using colour, so topics were coloured uniformly. Finally, the analysis resulted in 12 subcategories, which were further reflected in four main categories with three subcategories each (Fig. 4).

All the authors have had insight into all parts of the study and approved the final version of the manuscript. CB is responsible for the larger research project and, therefore, conducted the ethics application, recruitment and data collection. ÅGL and CB coded and stored the data. ÅGL performed the quantitative analysis and regularly coordinated with CB. The qualitative analysis was performed during recurring discussions in the author group, with the initial analysis conducted by ÅGL. The author group (ÅGL, ML, MH, ST and CB) comprised researchers representing various professions (district nurse, critical care nurse and midwife) and academic degrees (PhD and associate professor).

## Results

### Quantitative results

#### Relationship stability among mothers and partners at T1-T6

In total, 381 participants (mothers *n* = 242; partners *n* = 139) completed T6 and were eligible for analysis. The respondents’ characteristics included first-time mothers and their partners, of which 20% were on parental leave and 76% were employed four years after childbirth. Of these, 264 (94%) parental couples out of 281 had stable relationships four years after childbirth, 96% were born in Sweden, and 71% had an additional child four years after childbirth. The mean age at the initial birth was 28 for mothers and 30 for partners. Overall, the descriptive statistics indicate that the mean SOC, MSPSS and QDR scores were highest at T2 and lowest at T6 for both mothers and partners.

#### Simple logistic regression analysis

The SOC index among mothers at six months (T3) and at four years (T6) was associated with higher odds of relationship stability, which is presented in Table 2. In addition, for partners, a decrease in SOC between one week (T2) and two years (T5) after childbirth resulted in lower odds of relationship stability when analysing the mean indexes (SOC, MSPSS and QDR) in the simple regression analysis at different time points, T2 and T3, as well as T2 and T4. Partners with a higher mean MSPSS index had significantly higher odds of relationship stability four years (T6) after childbirth (Table 2). Furthermore, partners who perceived more support from their families at one week (T2), six months (T3) and from significant others at six months (T3) and one year (T4) had higher odds of relationship stability four years (T6) after childbirth (Supplementary file Table 2). An increase in MSPSS among mothers between the time points T2 and T3, T2 and T4, and T2 and T5 was associated with higher odds of relationship stability (Table 2). Additionally, mothers who reported a higher perceived quality of dyadic relationships at six months (T3), two years (T5) and four years (T6) had higher odds of relationship stability four years after childbirth (T6). See Table 2. Likewise, partners who reported higher-quality dyadic relationships two years (T5) after childbirth had higher odds of relationship stability four years (T6) after childbirth.

Significant results showed that higher perceived dyadic satisfaction (T4) and higher perceived dyadic sensuality (T5) were associated with relationship stability four years (T6) after childbirth for mothers and partners (Supplementary file Table 2). In addition, there were lower odds of relationship stability among mothers who reported a decrease in the perceived quality of their dyadic relationships between one week (T2) and six months (T3), as well as between one week (T2) and one year (T4) after childbirth. A better perceived good economic situation for mothers and partners at T2, for mothers at T4 and partners at T5 was associated with higher odds for relationship stability four years (T6) after childbirth. Likewise, those partners who answered that nothing happened that affected their well-being negatively at two years (T5) and four years (T6) had higher odds of relationship stability four years (T6) after childbirth.

#### Multiple logistic regression analysis

The results from the multiple logistic regression analysis illustrated that, for mothers, a better perceived financial situation one week (T2) after childbirth was related to higher odds of relationship stability (Table 3). Partners with better perceived social support from family one week (T2) after childbirth had higher odds of relationship stability four years (T6) after childbirth. Furthermore, the odds of relationship stability were related to the SOC index at four years (T6), with the perceived quality of dyadic relationships (QDR), though with a slightly lower odds ratio. Perceived dyadic satisfaction was a notable factor at (T3) and one year (T4).

#### Generalised Estimating Equations (GEE)

While the simple logistic regressions showed varying significance at different time points (Table 2), the overall longitudinal GEE model demonstrated significant associations between MSPSS (OR = 0.99, 95% CI [0.99, 1.00], *p* = .01) and QDR (OR = 1.02, 95% CI [1.01, 1.04], *p* = .004) and the outcome across the study period (Table 4). SOC was not significantly associated with the outcome. Among the background variables, years of education was significantly associated with the outcome.

#### Kaplan–Meier survival analysis

The Kaplan–Meier Survival Analysis revealed that the proportion of parents reporting no negative experiences (of negatively affected well-being) decreased over time. Specifically, at one week (T2) after childbirth, 92% of the respondents reported no adverse effects. This percentage remained relatively stable (89%) at six months but declined to 75% at one year (T4) and 66% at two years (T5). By four years (T6) after childbirth, six out of 10 parents (59%) reported that nothing had occurred that negatively impacted their well-being; consequently, for 41%, something had affected their well-being (Fig. 2). The Kaplan–Meier analysis of parents with relationship stability versus those who were separated revealed that until one year postpartum, parents who separated also reported experiencing negative well-being to a lesser degree than those who had relationship stability. However, from two (T5) to four (T6) years postpartum, the trend reversed, and those who were separated reported experiencing negative well-being to a greater extent (Fig. 3).

At one week (T2), 7.6% had experienced negatively affected well-being, and four years (T6) after childbirth, four out of 10 parents stated that something had affected their well-being negatively.

### Qualitative results

#### Description of what happened that affected well-being negatively (T2–T6)

Between 29 and 109 survey responses were available at T2–T6 to analyse participants’ written responses to whether their well-being was harmed by something that had happened. The qualitative analysis of what happened that affected participants’ well-being negatively resulted in four main categories, denoted as follows: changes in their own health status and well-being following childbirth; illness, death and strained relationships among family and friends; the unpredictability of everyday life requiring adaptability and multidimensional vulnerability in the parental-couple relationship (Fig. 4).

#### Changes in health status and well-being following childbirth

Parents described deteriorated health and long-lasting health problems in the four years after childbirth, including physical and mental health. Parents recounted how these problems affected their well-being due to, for example physical issues, such as disc herniation, fibromyalgia and joint pain. Mothers often described their physical health problems in terms of postpartum injuries; some mentioned that sphincter ruptures had inhibited their daily lives. Others commented on being incorrectly sewn, followed by painful intercourse (i.e. dyspareunia) and toilet visits. ‘I was sewn incorrectly in the genitals, so I have major pains with my sex life, and even during toilet visits, I can get pain in the lower abdomen, which really disturbs my everyday life’ (Mother, T3). Parents described mental health problems as the blues, confirmed depression and sick leave due to fatigue syndrome and stress following childbirth. ‘I developed a stress reaction/[or] exhaustion syndrome due to a stressful period of work, commuting, having small children, building a house and a cohabitant [partner] being away, which is why I am currently on sick leave’ (Mother, T5). Miscarriages and aborted pregnancies affected the well-being of both parents. Parents also described the birth of an additional child as negatively affecting their well-being: ‘I became a father of two against my will’ (Partner, T6).

Moreover, parents expressed the realisation that they did not feel well physically or mentally and, when seeking professional support from a child healthcare psychologist or physician, sometimes experienced not being taken seriously or receiving satisfactory care. Parents remarked that one family member’s affected well-being impacted the whole family’s mood. Correspondingly, parents mentioned that the health problems of their children or older siblings with neuropsychiatric disorders sometimes led to stressful situations in the family.

#### Illness, death and strained relationships among family and friends

Parents’ well-being was also affected by their surrounding family and friends. Parents experienced family members’ illnesses and deaths as affecting their well-being and causing strained and disharmonious relationships. Parents described how their parents, grandparents, mothers-in-law and siblings were seriously physically ill, had died or were experiencing difficult times in their lives: ‘My stepdad passed away suddenly from cancer who was like an extra grandfather to our four-year-old child. It has been heavy. My family has, overall, been through tough things for the last two years’ (Mother, T6). Some parents also wrote that their parents or friends had committed suicide. Parents described the mental illness of their parents and losing contact with family members due to the situation or major family conflicts and quarrels with parents. Similarly, parents mentioned friends reacting indifferently to having a child: ‘A close friend reacted poorly to me having a baby, so now we have no contact’ (Mother, T3). Some partners had children from a previous relationship, and custody disputes affected their well-being.

#### The unpredictability of everyday life requires adaptability

Everyday family life requires parental couples to make decisions, establish priorities and consequently enact changes due to their workplace, choice of residence and adjustments in a changing and often strained financial situation: ‘The partner started studying in [city name] and commutes weekly. This causes me to be alone a lot, and our personal finances deteriorate’ (Mother, T2). The parents also described involuntary relocations for employment or a new job. Parents in the current study mentioned housing situations, such as building, maintaining and moving to a house, negatively affecting their well-being. For some parental couples, this meant daily commutes for work and weekly commutes due to living in different towns during the weekdays or even living on other continents for months: ‘The partner was quickly informed about serving abroad and has now gone to [another continent] to be there for six months’ (Mother, T4). When parents were separated in daily life, the parents who stayed with the child spent much time on their own, which led to loneliness. Hence, career advancement was reported to be followed by an increased commitment to work and less time for family life: ‘I have said yes to too much that has taken time out of my private life since I started working full time again and felt the stress of not being able to keep up with the family and our new house’ (Partner, T4). Parents also described life as a ‘puzzle’ when both parents started working, even working full-time and studying part-time. These major life changes often co-occurred after childbirth and could result in overwhelm.

#### Multidimensional vulnerability in the parental-couple relationship

Parents expressed strained parental-couple relationships, poor communication and a lack of love. Some admitted that their partners were engaged in infidelity, which resulted in the parental couple’s separation. Wanting different things within the parental couple was noted, as well as not wanting to do the same things as before their pregnancy and less time for physical activity and sleep: ‘Less time for physical activity and sleep, which naturally affects well-being negatively’ (Partner, T3). Parents described a lack of sleep sometimes followed by extreme tiredness, which interfered with their well-being and the couple’s relationship: ‘I am worn out from a lack of sleep and from the challenging adjustment for the [my] spouse to have a baby’ (Mother, T3). A lack of time was also mentioned: ‘Time is very scarce to catch up with one’s recovery and contact with the [my] partner without the children. It wears hard on [my] well-being’ (Partner, T6). The partners also described a lack of time alone and feeling powerless.

Furthermore, partners were prepared to help but considered the mother more important regarding childcare. Partners harboured opinions regarding the need for an equal division of childcare and household labour to increase both parents’ individual free time: ‘Less free time. Powerlessness, as I can imagine, that I have to help but may not be able to do much during the kid’s first time, which has resulted in none of us getting any free time. You might have been able to divide it up better there, but the mother is, of course, more important in the beginning’ (Partner, T3).

Additionally, parents described increased responsibility for themselves, their partners and their children. These parents mentioned feeling more emotions than before their children were born.

## Discussion

The present study explored parents’ well-being and relationship stability from pregnancy to four years after childbirth by integrating quantitative and qualitative findings. Overall, the results show that both relational stability and parental well-being are shaped by dynamic and interrelated processes over time. A key finding was that a stronger sense of coherence (SOC) was associated with higher odds of relationship stability at four years postpartum, whereas a decline in SOC between one week (T2) and two years after childbirth (T5) was linked to lower relationship stability. These findings position SOC as a dynamic resource that evolves during the transition to parenthood and plays a central role in long-term relationship outcomes.

The qualitative findings deepen this understanding by illustrating how parental well-being and relational stability are embedded in everyday life experiences and shaped by life events and relational challenges, such as bereavement or relationship strain. In this context, vulnerability emerges not as a deviation from normality, but as an inherent aspect of the transition to parenthood [48].

The quantitative findings at the four-year follow up indicated that higher maternal SOC was associated with increased odds of relationship stability, whereas lower partner SOC was associated with reduced odds of maintaining the relationship. By explicitly integrating these metrics with our qualitative findings, we can better understand this decline. The theme ‘The unpredictability of everyday life requires adaptability’ illustrates how challenges related to employment, housing and loneliness negatively affected parents’ well-being and the ability to manage everyday life. Similarly, the financial strain described by the participants in the qualitative results corresponds with the quantitative finding that a more favourable perceived economic situation was associated with greater odds for relationship stability at four years.

The quantitative analyses also demonstrated that greater levels of perceived social support among mothers were associated with increased odds of relationship stability. This finding is reflected in the qualitative results, where participants described a challenging time period characterised by strained relationships with family members and friends. Together, these findings suggest that social support may serve as an important resource for sustaining parental relationships during the transition to parenthood and the years that follow.

Furthermore, the quantitative results showed that decreases in mothers´ perceived quality of the dyadic relationship during the first year after childbirth were associated with lower odds for relationship stability. This qualitative theme ‘Multidimensional vulnerability in the parental-couple relationship’ provides important contextual insights into this association. Parents described strained parental-couple relationships, sleep deprivation, and heightened emotional responses as significant challenges during the early parenting period. These findings complement to the qualitative results, which demonstrated that higher levels of dyadic satisfaction and dyadic sensuality were associated with greater odds for relationship stability at four years. Taken together, the results underscore that maintaining a stable parental relationship requires ongoing effort, attention, and mutual commitment – particularly during the transition to parenthood and the subsequent years of family life.

Taken together, the findings suggest that fluctuations in SOC may reflect parents’ capacity to make sense of and manage such challenges, thereby influencing both individual well-being and relationship stability over time. This integrated perspective highlights the importance of understanding the transition to parenthood as a dynamic process in which existential, relational and contextual factors continuously interact [49].

This interpretation aligns with previous research suggesting continuity between antenatal and postnatal SOC [36], as well as studies showing that becoming a parent can enhance a sense of meaning and purpose [5, 36]. From a salutogenic perspective, parents who perceive their situation as comprehensible and manageable are better equipped to handle the uncertainty and demands of early parenthood [50]. Therefore, a strong family SOC may support adaptation following periods of stress and crisis [45].

Previous research also indicates that mothers’ emotional satisfaction is linked to their partner’s participation in domestic responsibilities [49], and that many mothers seek opportunities to share experiences and receive practical and material support, including help with household tasks [29].

Changes in intimacy also appear relevant. Higher levels of perceived sensuality were, in this study, associated with greater relationship stability, indicating that emotional and physical closeness may play a protective role [51]. Given that intimacy often changes after childbirth, reduced closeness may signal a need for support. Accordingly, interventions that, for example strengthen communication [10] and address intimacy could be beneficial. This corroborates research linking sensuality, curiosity, imagination and emotional connection with sexual satisfaction [52, 53].

Sleep deprivation and communication difficulties emerged as additional stressors affecting well-being. These challenges, combined with major life adjustments such as changes in health, work and relationship dynamics, place considerable demands on parents’ resources. Indeed, as Barimani et al. stated, being unprepared may hinder the transition to parenthood [54]. A reflection is that individualised support from healthcare professionals may help parents navigate these changes more effectively, which corroborates Larsson et al. [55], who propose that individualised support promotes clearer communication of expectations and needs. Involving both parents and encouraging equal participation may also strengthen the development of both the parental role and the relationship [55].

Social support was another important factor. Higher perceived support, particularly among partners, was associated with greater relationship stability. Increases in mothers’ perceived support over time were similarly linked to more stable relationships. These findings reinforce the role of social support as a resource that enhances the manageability and meaning of parenthood [50]. They also align with previous studies showing that a lack of support and social isolation can increase stress and the risk of separation, particularly among fathers [56, 57].

The Swedish context may provide additional insights into these patterns. Around one year postpartum, parents often alternate parental leave, which may increase fathers’ involvement in childcare. Greater engagement during early childhood has been associated with stronger parental roles and may influence both well-being and relationship dynamics [58].

Taken together, these findings suggest that the transition to parenthood requires substantial support at multiple levels. Both parents must be included [24] yet previous research indicates that fathers are often marginalised in antenatal and postnatal care [59]. Addressing this gap may enhance both individual well-being and relationship outcomes. Encouraging reflection on the existential aspects of parenthood – such as meaning, identity and coping with uncertainty – may also be valuable [7, 60]. Healthcare professionals, particularly midwives and child health care nurses, are well positioned to facilitate such discussions and provide health-promotive support [61].

Overall, this study underscores the importance of considering both individual and relational processes in understanding parents’ well-being and relationship stability over time.

### Implications

The implications for practice are that it is important to support parental well-being, a sense of coherence, social support and the quality of dyadic relationships to handle potential decreases in well-being and increase the odds of a stable relationship. Knowing that well-being decreases and that SOC is changing and associated with relationship stability, it is crucial for healthcare professionals, such as midwives and CHC nurses, to support parents’ well-being by asking parents questions such as ‘How do you feel about being a parent?’, ‘What makes you feel good about being a parent?’, ‘How do you experience your daily life?’, ‘When do you experience feelings of meaningfulness?’ and ‘How do you handle being a parent?’

Ultimately, this implies that midwives and CHC nurses can make efforts to strengthen parents’ existential well-being during this vulnerable period of life. By understanding gender equality and participation, CHC nurses can strengthen the role of non-birthing parents in the parental role. Postpartum physical recovery can last long and needs attention. Furthermore, the importance of social support should be emphasised, and more information about changes in the parental-couple’s relationship should be generated to augment the importance of the social context. Parents need support regarding how parenthood affects their relationship. In addition, existential aspects must be included in the healthcare professional’s support during pregnancy, throughout parenthood and for the first four years after childbirth. Further studies can examine how relationship stability may fluctuate over longer time periods (e.g. eight or 10 years after childbirth, etc.).

### Limitations

This study included only couples who have given birth to their first child (first-time mothers and their partners). The results may differ for parents with additional or multiple births (e.g. twins and triplets). Furthermore, most couples in this study were the opposite sex. Therefore, the findings may not generalise to same-sex couples or single mothers. Understanding and speaking the Swedish language was required for participation. In 2021, Sweden had just over two million inhabitants who were born abroad, which is 20% of the population [29]. Some of these people do not speak Swedish. This sample was not fully representative, which could interfere with the generalisability of the results. Due to the explorative nature of this study and the various statistical tests performed, significant p-values should not be regarded as conclusive evidence until repeated in a confirmatory study.

The open-ended question about parents’ well-being was posed from a negative perspective, which could be considered a limitation because the answers described the factors that contributed to relationship stability. The qualitative question on well-being had a word limit of 80 words, culminating in some interrupted responses that potentially impacted trustworthiness. The relatively many questions in the questionnaire also could be a limitation because the parents’ full attention, which was needed to complete the questionnaire, could have been disrupted. All the answers to the open-ended questions were analysed; however, data saturation was not achieved, which can be a limitation. A pilot study was performed before the larger research project, demonstrating that parents perceived the questionnaires as easy to respond to and understand. Additionally, to gain a deeper understanding of how parenthood affects an individual’s adult life, more research is needed on individual and parental-couple transitions [25].

The study’s mixed-method design was based on the idea that a better understanding is provided by combining quantitative and qualitative approaches, a technique particularly salient today, given the complexity of the problems that must be addressed [62].

## Conclusion

The indications found in this study are the following: Parents’ abilities to maintain a sense of coherence, access and receive social support and maintain a high-quality parental-couple relationship are associated with relationship stability four years after childbirth. Maintaining relationship stability is an ongoing process in which parents must navigate both the social contextual conditions and the existential changes that parenthood entails. Both individual and relational processes should be considered in efforts to support parents’ well-being and long-term relationship stability.

## Supplementary Information

Supplementary Material 1.

## Data Availability

The data set analysed during the current study is available from the corresponding author on reasonable request.

## Abbreviations

CHC: Child Health Care
DAS: Dyadic Adjustment Scale
EvaSys: Evaluation System
MSPSS: Multidimensional Scale of Perceived Social Support
QDR: Quality of the Dyadic Relationship
SOC: Sense of Coherence
SPSS: Statistical Package for the Social Sciences
