# Supplementary material for: Parental-couple well-being and relationship stability four years following childbirth: a mixed-methods study
Source: Reprod Health. 2026 Oct 9;23:183. doi: 10.1186/s12978-026-02469-9 (PMC13660268; doi:10.1186/s12978-026-02469-9)
Supplement: Supplementary file 1 [file 12978_2026_2469_MOESM1_ESM.docx]

|  | **TABLE 2.** Simple Logistic Regression of Relationship Stability T6, at Different Time Points (T1-T6). | | | |
| --- | --- | --- | --- | --- |
|  | | **Total** | **Mothers** | **Partners** |
|  | | **OR (95 % CI)** | **OR (95 % CI)** | **OR (95 % CI)** |
| Participant, *n* | | 381 | 242 | 139 |
| Age at birth of the first child | | 1.05 (0.94-1.18) | 1.10 (0.93–1.29) | 1.00 (0.86–1.14) |
| Education T6 (yrs) | | **1.23** (1.09-1.39)*** | **1.18** (1.03–1.36)* | **1.46** (1.09–1.97)* |
| Country of birth of the  participant T6 (Sweden) | | 1.21 (0.57-2.57) | 1.40 (0.66–3.00) | a |
| Marital status T6 range 1–4  (married) | | a | a | a |
| Occupation T6 | |  |  |  |
| Parental-leave T6 | | 4.88 (0.64-37.14) | 4.29 (0.55–33.67) | a |
| Employee T6 | | 0.82 (0.26-2.52) | 0.64 (0.17–2.38) | 1.46 (0.16–13.33) |
| Studying T6 | | 1.34 (0.17-10.45) | 0.27 (0.16–10.33) | a |
| Sick-leave T6 | | **0.14** (0.03-0.77) * | 0.27 (0.03–2.49) | **0.04** (0.002–0.70) * |
| Unemployed T6 | | a | a | - |
| Other T6 | | a | a | a |
| Perceived economy | |  |  |  |
| T1 | | 2.45 (0.95-6.30) | 2.78 (0.89–8.67) | 1.72 (0.29–10.20) |
| T2 | | **3.82** (1.91-7.61)*** | **3.53** (1.49–8.40)** | **4.34** (1.38–13.65)* |
| T3 | | 1.44 (0.78-2.65) | 1.87 (0.84–4.18) | 0.93 (0.30–2.88) |
| T4 | | 1.86 (0.98-3.54) | **2.49** (1.09–5.70)* | 1.02 (0.32–3.26) |
| T5 | | **2.45** (1.28-4.69)** | 2.07 (0.89–4.81) | **3.15** (1.12–8.82)* |
| T6 | | **1.83** (1.06-3.16)* | 1.89 (0.996–3.59) | 1.64 (0.58–4.67) |
| Planned pregnancy 2 years ago T5 (yes) | | 0.82 (0.19-3.58) | 0.77 (0.10–6.08) | 0.97 (0.12–8.00) |
| Additional births, range 1–3 (1, 2, and >2 births) | |  |  |  |
| T4 | | a | a | a |
| T5 | | 2.74 (0.56-13.53) | 1.88 (0.34–10.46) | a |
| T6 | | **5.96** (1.79-19.82)** | **4.33** (1.13–16.51)* | a |
| Length of couple relationship T6 | | 1.33 (0.96-1.83) | 1.32 (0.96–1.83) | a |
| If something occurred that affected every-day well-being negatively T2–T6 (no) | |  |  |  |
| T2 | | 0.69 (0.12-4.12) | 0.90 (0.16–5.02) | a |
| T3 | | 1.28 (0.49-3.33) | 1.87 (0.67–5.18) | a |
| T4 | | 1.47 (0.65-3.32) | 1.80 (0.78–4.19) | a |
| T5 | | **2.24** (1.12-4.48)* | 1.72 (0.62–4.77) | **2.89** (1.07–7.81)* |
| T6 | | **2.01** (1.16-3.79) * | 1.73 (0.83–3.62) | **3.19** (1.11–9.12)* |
| Professional Preparatory  Support | |  |  |  |
| Attending Professional  Preparatory Support with Partner T5 (yes) | | 0.51 (0.11-2.41) | 0.50 (0.10-2.49) | a |
| Experienced Professional Preparatory Support T3 | | **2.21** (1.09-4.49)* | 1.96 (0.84–4.57) | 3.11 (0.80–12.02) |
| **SOC-13** | |  |  |  |
| **SOC-13 index T1** | | 1.02 (0.97-1.08) | 1.03 (0.96–1.10) | 1.01 (0.91–1.11) |
| Comprehensibility T1 | | 1.04 (0.92-1.17) | 1.05 (0.91–1.22) | 1.01 (0.81–1.27) |
| Manageability T1 | | 1.07 (0.92-1.24) | 1.08 (0.90–1.29) | 1.04 (0.77–1.40) |
| Meaningfulness T1 | | 1.08 (0.91-1.28) | 1.12 (0.91–1.37) | 0.995 (0.72–1.37) |
| **SOC-13 index T2** | | 1.01 (0.96-1.06) | 1.01 (0.96–1.07) | 1.00 (0.92–1.10) |
| Comprehensibility T2 | | 1.01 (0.91-1.11) | 1.00 (0.89–1.13) | 1.01 (0.85–1.21) |
| Manageability T2 | | 1.05 (0.93-1.19) | 1.06 (0.91–1.22) | 1.05 (0.84–1.30) |
| Meaningfulness T2 | | 1.05 (0.91-1.21) | 1.02 (0.86–1.22) | 1.11 (0.86–1.44) |
| **SOC-13 index T3** | | 1.03 (0.996-1.07) | **1.05** (1.00–1.09)* | 1.00 (0.94–1.07) |
| Comprehensibility T3 | | 1.03 (0.95-1.11) | 1.06 (0.96–1.17) | 0.95 (0.80–1.12) |
| Manageability T3 | | **1.13** (1.02-1.24)* | **1.17** (1.04–1.32)** | 1.04 (0.86–1.27) |
| Meaningfulness T3 | | **1.12** (1.00-1.25)* | 1.15 (0.995–1.32) | 1.08 (0.90–1.30) |
| **SOC-13 index T4** | | 1.02 (0.99-1.06) | 1.03 (0.99–1.07) | 0.99 (0.92–1.07) |
| Comprehensibility T4 | | 1.05 (0.96-1.13) | 1.06 (0.97–1.17) | 0.98 (0.82–1.17) |
| Manageability T4 | | 1.09 (0.99-1.20) | 1.12 (0.997–1.25) | 0.996 (0.80–1.24) |
| Meaningfulness T4 | | 1.01 (0.89-1.15) | 1.01 (0.88–1.18) | 0.99 (0.77–1.27) |
| **SOC-13 index T5** | | 1.02 (0.98-1.07) | 1.00 (0.95–1.06) | 1.07 (0.99–1.15) |
| Comprehensibility T5 | | 1.02 (0.93-1.13) | 0.95 (0.83–1.09) | 1.15 (0.99–1.35) |
| Manageability T5 | | 1.09 (0.97-1.23) | 1.10 (0.95–1.28) | 1.08 (0.89–1.31) |
| Meaningfulness T5 | | 1.03 (0.89-1.18) | 0.996 (0.83–1.20) | 1.07 (0.86–1.33) |
| **SOC-13 index T6** | | **1.05** (1.02-1.09)** | **1.06** (1.02–1.11)** | 1.03 (0.96–1.09) |
| Comprehensibility T6 | | **1.09** (1.01-1.18)* | **1.11** (1.01–1.22)* | 1.03 (0.89–1.20) |
| Manageability T6 | | **1.21** (1.10-1.33)*** | **1.30** (1.14–1.48)*** | 1.08 (0.90–1.28) |
| Meaningfulness T6 | | 1.09 (0.98-1.22) | 1.10 (0.96-1.25) | 1.09 (0.89–1.33) |
| **Mean** | |  |  |  |
| Mean SOC-13 index T2-T3 | | 1.02 (0.97-1.07) | 1.03 (0.98-1.09) | 0.998 (0.91-1.09) |
| Mean SOC-13 index T2-T4 | | 1.02 (0.97-1.07) | 1.03 (0.98-1.09) | 0.99 (0.90-1.09) |
| Mean SOC-13 index T2-T5 | | 1.02 (0.96-1.08) | 1.02 (0.96-1.09) | 1.02 (0.91-1.14) |
| Mean SOC-13 index T2-T6 | | 1.02 (0.97-1.08) | 1.02 (0.96-1.09) | 1.01 (0.90-1.14) |
| **Change** | |  |  |  |
| Change SOC-13 index T2-T3 | | 0.98 (0.92-1.04) | 0.97 (0.91-1.03) | 1.03 (0.88-1.19) |
| Change SOC-13 index T2-T4 | | 0.99 (0.93-1.06) | 0.97 (0.91-1.03) | 1.10 (0.96-1.26) |
| Change SOC-13 index T2-T5 | | 0.96 (0.90-1.03) | 1.01 (0.92-1.09) | **0.76** (0.60-0.96)* |
| Change SOC-13 index T2-T6 | | 1.05 (0.997-1.10) | 1.06 (0.995-1.12) | 1.03 (0.93-1.14) |
| **MSPSS** | |  |  |  |
| **MSPSS index T1** | | 0.996 (0.94-1.06) | 0.92 (0.81–1.05) | 1.06 (0.98–1.14) |
| Family T1 | | 1.02 (0.91-1.14) | 0.91 (0.72–1.14) | 1.13 (0.97–1.31) |
| Friends T1 | | 0.93 (0.78-1.10) | 0.81 (0.61–1.09) | 1.06 (0.87–1.29) |
| Significant Others T1 | | 1.04 (0.86-1.25) | 0.89 (0.59–1.32) | 1.17 (0.93–1.48) |
| **MSPSS index T2** | | **1.05** (1.00-1.11)* | 1.02 (0.94–1.11) | **1.11** (1.02–1.21)* |
| Family T2 | | **1.13** (1.02-1.25)* | 1.04 (0.89–1.21) | **1.31** (1.09–1.56)** |
| Friends T2 | | 1.07 (0.96-1.19) | 1.04 (0.89–1.21) | 1.13 (0.96–1.33) |
| Significant Others T2 | | 1.09 (0.89-1.34) | 0.98 (0.62–1.55) | 1.22 (0.92–1.63) |
| **MSPSS index T3** | | 1.02 (0.98-1.07) | 0.98 (0.91–1.05) | **1.09** (1.01–1.17)* |
| Family T3 | | 1.06 (0.97-1.16) | 0.98 (0.85–1.13) | **1.17** (1.03–1.34)* |
| Friends T3 | | 1.02 (0.93-1.13) | 0.95 (0.82–1.10) | 1.15 (0.98–1.35) |
| Significant Others T3 | | 1.06 (0.93-1.21) | 0.90 (0.67–1.20) | **1.27** (1.02–1.57)* |
| **MSPSS index T4** | | 1.01 (0.97-1.05) | 0.97 (0.91–1.03) | 1.06 (0.998–1.12) |
| Family T4 | | 1.03 (0.95-1.13) | 0.99 (0.88–1.12) | 1.12 (0.97–1.29) |
| Friends T4 | | 0.98 (0.88-1.08) | 0.90 (0.78–1.05) | 1.11 (0.95–1.30) |
| Significant Others T4 | | 1.04 (0.93-1.17) | 0.88 (0.69–1.12) | **1.21** (1.03–1.41)* |
| **MSPSS index T5** | | 1.00 (0.96-1.05) | 0.92 (0.83–1.02) | 1.06 (0.997–1.13) |
| Family T5 | | 0.98 (0.87-1.10) | 0.85 (0.67–1.07) | 1.11 (0.95–1.29) |
| Friends T5 | | 1.01 (0.90-1.13) | 0.86 (0.69–1.06) | 1.17 (0.99–1.39) |
| Significant Others T5 | | 1.03 (0.91-1.18) | 0.73 (0.45–1.16) | 1.16 (0.996–1.35) |
| **MSPSS index T6** | | 1.02 (0.99-1.06) | 1.01 (0.97–1.05) | **1.06** (1.00–1.12)* |
| Family T6 | | 1.07 (0.995-1.15) | 1.05 (0.95–1.15) | 1.13 (0.99–1.29) |
| Friends T6 | | 1.04 (0.96-1.13) | 0.995 (0.89–1.11) | 1.13 (0.98–1.30) |
| Significant Others T6 | | 1.06 (0.96-1.16) | 1.00 (0.88–1.14) | 1.15 (0.99–1.34) |
| **Mean** | |  |  |  |
| Mean MSPSS index T2-T3 | | 1.03 (0.97-1.09) | 0.96 (0.86-1.06) | **1.10** (1.01-1.20)* |
| Mean MSPSS index T2-T4 | | 1.02 (0.97-1.07) | 0.95 (0.86-1.05) | **1.09** (1.01-1.17)* |
| Mean MSPSS index T2-T5 | | 1.01 (0.95-1.08) | 0.95 (0.85-1.06) | 1.07 (0.99-1.16) |
| Mean MSPSS index T2-T6 | | 1.01 (0.96-1.08) | 0.95 (0.86-1.06) | 1.07 (0.99-1.16) |
| **Change** | |  |  |  |
| Change MSPSS index T2-T3 | | **1.10** (1.02-1.19)* | **1.17** (1.05-1.30)** | 0.995 (0.88-1.12) |
| Change MSPSS index T2-T4 | | 1.06 (0.99-1.14) | **1.10** (1.00-1.20)* | 0.998 (0.90-1.11) |
| Change MSPSS index T2-T5 | | **1.08** (1.00-1.17)* | **1.12** (1.02-1.23)* | 1.02 (0.91-1.15) |
| Change MSPSS index T2-T6 | | 0.96 (0.91-1.02) | 0.94 (0.87-1.01) | 1.01 (0.92-1.10) |
| **QDR36** | |  |  |  |
| **QDR36 index T1** | | 1.26 (0.89-1.80) | 1.34 (0.87–2.05) | 1.09 (0.55–2.15) |
| Dyadic Consensus T1 | | 1.37 (0.32-5.87) | 1.53 (0.22–9.41) | 1.21 (0.10–14.45) |
| Dyadic Cohesion T1 | | **2.61** (1.08-6.36)* | 2.60 (0.94–7.17) | 2.60 (0.36–18.74) |
| Dyadic Satisfaction T1 | | 3.50 (0.83-14.87) | 4.23 (0.72–24.81) | 2.40 (0.18–31.72) |
| Dyadic Sensuality T1 | | 2.25 (0.82-6.17) | 2.85 (0.87–9.32) | 1.46 (0.19–11.26) |
| Dyadic Sexuality T1 | | 0.87 (0.29-2.60) | 1.06 (0.32–3.49) | 0.45 (0.04–5.24) |
| **QDR36 index T2** | | 1.14 (0.86-1.51) | 1.11 (0.78–1.59) | 1.19 (0.75–1.89) |
| Dyadic Consensus T2 | | 1.67 (0.70-4.01) | 1.34 (0.44–4.07) | 3.46 (0.51–23.56) |
| Dyadic Cohesion T2 | | 1.93 (0.92-4.03) | 1.65 (0.69–3.95) | 2.69 (0.67–10.73) |
| Dyadic Satisfaction T2 | | 2.91 (0.88-9.69) | 2.76 (0.60–12.73) | 3.18 (0.45–22.45) |
| Dyadic.Sensuality T2 | | 1.59 (0.65-3.91) | 1.60 (0.49–5.19) | 1.58 (0.39–6.36) |
| Dyadic Sexuality T2 | | 0.90 (0.37-2.19) | 1.28 (0.42–3.93) | 0.48 (0.10–2.33) |
| **QDR36 index T3** | | **1.29 (**1.07-1.55)** | **1.37** (1.08–1.74)** | 1.17 (0.87–1.58) |
| Dyadic Consensus T3 | | **2.55** (1.26-5.15)** | **3.05** (1.36–6.86)** | 1.50 (0.33–6.84) |
| Dyadic Cohesion T3 | | 1.77 (0.96-3.27) | 1.69 (0.77–3.73) | 1.98 (0.74–5.32) |
| Dyadic Satisfaction T3 | | **4.25** (1.92-9.41)*** | **6.15**(2.11–17.93)*** | 2.59 (0.71–9.41) |
| Dyadic Sensuality T3 | | 1.22 (0.64-2.34) | 1.49 (0.64–3.49) | 0.93 (0.33–2.65) |
| Dyadic Sexuality T3 | | 1.84 (0.92-3.64) | 1.87 (0.75–4.65) | 1.75 (0.62–4.96) |
| **QDR36 index T4** | | **1.21** (1.02-1.43)* | 1.18 (0.96–1.44) | 1.29 (0.93–1.78) |
| Dyadic Consensus T4 | | **2.24** (1.06-4.74)* | 2.15 (0.92–5.06) | 2.54 (0.52–12.34) |
| Dyadic Cohesion T4 | | 1.56 (0.87-2.78) | 1.59 (0.82–3.08) | 1.44 (0.42–4.93) |
| Dyadic Satisfaction T4 | | **3.43** (1.69-6.98)*** | **2.85** (1.25–6.49)* | **7.24** (1.48–35.38)* |
| Dyadic Sensuality T4 | | 1.35 (0.75-2.43) | 1.30 (0.63–2.67) | 1.44 (0.51–4.05) |
| Dyadic Sexuality T4 | | 1.62 (0.83-3.19) | 1.39 (0.59–3.28) | 2.11 (0.70–6.41) |
| **QDR36 index T5** | | **1.29** (1.08-1.55)** | **1.26** (1.01–1.56)* | **1.41** (1.00-1.99)* |
| Dyadic Consensus T5 | | **2.73** (1.23-6.08)* | **2.75** (1.08–6.98)* | 2.96 (0.55–16.08) |
| Dyadic Cohesion T5 | | 1.28 (0.64-2.55) | 0.80 (0.32–2.02) | 3.26 (0.92–11.59) |
| Dyadic Satisfaction T5 | | **3.19** (1.51-6.74)** | **3.25** (1.35–7.78)** | 3.61 (0.77–16.95) |
| Dyadic Sensuality T5 | | **2.86** (1.50-5.46)*** | **2.40** (1.8-5.35)* | **4.18** (1.29–13.60)* |
| Dyadic Sexuality T5 | | **2.30** (1.03-5.17)* | **2.89** (1.03–8.12)* | 1.62 (0.44–6.03) |
| **QDR36 index T6** | | **1.27** (1.08-1.51)** | **1.37** (1.14–1.66)*** | 0.59 (0.29–1.19) |
| Dyadic Consensus T6 | | **3.78** (1.81-7.91)*** | **3.99** (1.81–8.81)*** | 1.38 (0.11–17.83) |
| Dyadic Cohesion T6 | | **2.41** (1.28-4.52)** | **2.69** (1.36–5.34)** | 0.77 (0.13–4.39) |
| Dyadic Satisfaction T6 | | **3.05** (1.49-6.24)** | **3.62** (1.63–8.04)** | 0.30 (0.02–6.15) |
| Dyadic Sensuality T6 | | **2.35** (1.34-4.14)** | **3.32** (1.66–6.62)*** | 0.11 (0.003–4.27) |
| Dyadic Sexuality T6 | | 0.86 (0.38-1.92) | 1.80 (0.76–4.28) | a |
| **Mean** | |  |  |  |
| Mean QDR36 index T2-T3 | | 1.16 (0.86-1.55) | 1.19 (0.80-1.77) | 1.11 (0.72-1.72) |
| Mean QDR36 index T2-T4 | | 1.21 (0.92-1.59) | 1.20 (0.84-1.73) | 1.21 (0.80-1.85) |
| Mean QDR36 index T2-T5 | | 1.32 (0.93-1.87) | 1.13 (0.71-1.81) | 1.79 (0.86-3.73) |
| Mean QDR36 index T2-T6 | | 1.48 (0.90-2.42) | 1.66 (0.87-3.17) | 1.29 (0.56-2.95) |
| **Change** | |  |  |  |
| Change QDR36 index T2-T3 | | 0.80 (0.60-1.06) | **0.71** (0.51-1.00)* | 1.01 (0.56-1.83) |
| Change QDR36 index T2-T4 | | **0.71** (0.55-0.92)** | **0.72** (0.53-0.96)* | 0.66 (0.36-1.19) |
| Change QDR36 index T2-T5 | | 0.74 (0.54-1.01) | 0.82 (0.56-1.20) | 0.56 (0.30-1.04) |
| Change QDR36 index T2-T6 | | 0.80 (0.55-0.15) | 1.03 (0.72-1.46) | 0.11 (0.01-1.28) |
|  | **Note:** OR–Values with a Significant P-value, Marked in Bold. *p-value ≤0.05; ** p-value ≤0.01; *** p-value ≤0.001; a= did not converge. | | | |
